# Bridging Access Gaps in Radiotherapy: Knowledge-Based Planning in a Resource-Constrained Clinical Setting

**DOI:** 10.7759/cureus.109306

**Published:** 2026-05-20

**Authors:** Milton E Ixquiac Cabrera, Erick O Montenegro, Matthew Schmidt, Baozhou Sun, James A Kavanaugh, Taoran Li, Angel Velarde, Vicky De Falla, Francisco Reynoso

**Affiliations:** 1 Physics, Escuela de Ciencias Fisicas y Matemáticas, Universidad de San Carlos de Guatemala, Guatemala City, GTM; 2 Radiotherapy, Liga Nacional Contra el Cáncer, Guatemala City, GTM; 3 Radiation Oncology, Washington University School of Medicine, St. Louis, USA; 4 Medical Physics, Baylor College of Medicine, San Antonio, USA; 5 Radiation Oncology, Mayo Clinic, Rochester, USA; 6 Radiation Oncology, University of Pennsylvania, Philadelphia, USA; 7 Oncology, Instituto Nacional de Cancerología (INCAN), Guatemala City, GTM; 8 Medical Science, Varian Medical Systems-Siemens Healthineers, Palo Alto, USA

**Keywords:** gynecological cancer, knowledge-based planning, low- and middle-income countries, radiotherapy, rapidplan, treatment planning efficiency, vmat

## Abstract

Background/purpose: Radiotherapy access in low- and middle-income countries (LMICs) is severely limited by shortages of trained personnel, high patient volumes, and large waiting times for manual treatment planning. Knowledge-based planning (KBP) has shown promise in high-income settings, yet its feasibility in resource-constrained environments remains underexplored. This study aimed to evaluate the feasibility of adapting a KBP model developed at a high-income institution for clinical use at Liga Nacional Contra el Cáncer (LNCC) in Guatemala for gynecological radiotherapy planning.

Materials and methods: A RapidPlan KBP model (Varian-Siemens Healthineers, Palo Alto, CA, USA) originally developed at Washington University in St. Louis using data from over 150 patients was adapted and augmented with 118 gynecological cases from the Instituto Nacional de Cancerología (INCAN), reflecting local contouring variability and clinical heterogeneity. Cases were categorized into three planning groups based on planning target volume (PTV) geometry: pelvic only, pelvic with inguinal nodes, and pelvic with inguinal and paraaortic nodes. A validation cohort of 25 gynecological cancer patients was used to compare KBP-generated plans against manual plans produced by four dosimetrists (two junior, two senior). Plans were evaluated using a dosimetric scorecard tool adapted from Varian Medical Affairs, assessing PTV coverage, organ at risk (OAR) sparing (bladder, rectum, and femoral heads), total planning time, plan quality efficiency score (total score/planning time), gamma passing rates (3%/2 mm), and monitor units.

Results: Overall plan quality was comparable between KBP-generated and manually generated plans across all planners, with no systematic differences in target coverage or OAR sparing. The KBP model produced the highest scores for rectal dose metrics compared to manual plans. All plans achieved similar gamma passing rates (mean 99.6%). The mean monitor unit was 264 ± 57 MU across all planners. Importantly, the KBP model significantly reduced plan creation time, resulting in higher plan quality efficiency scores, demonstrating that equivalent clinical quality can be achieved more rapidly.

Conclusion: KBP can be successfully adapted from a high-income institution for use in an LMIC setting, maintaining clinical plan quality while substantially reducing planning time. This approach supports increased throughput and more equitable access to high-quality radiotherapy in resource-constrained environments, even in the presence of contouring variability.

## Introduction

The incidence of cancer in Guatemala has risen in recent decades, primarily attributable to advancements in detection methods. However, access to cancer treatment, particularly radiotherapy, remains very limited. Guatemala, with a population nearing 20 million, has eight megavoltage (MV) therapy units and five brachytherapy devices, distributed across five radiotherapy facilities. Of the eight MV therapy units in the country, three are linear accelerators (LINACs) located in Liga Nacional Contra el Cáncer (LNCC). This equates to approximately 0.4 therapy units per million individuals, which is significantly less than the International Atomic Energy Agency's (IAEA) recommended standard of four therapy units per million people [1]. The LNCC is a nonprofit organization established in 1953 to coordinate cancer prevention and treatment nationwide [2]. Its legal framework and operational objectives are defined in its constitutive statutes [3]. LNCC serves as a specialized facility for cancer treatment and remains the only hospital in Guatemala to offer comprehensive services, including surgery, chemotherapy, and radiotherapy. The Guatemalan government has established an agreement with LNCC to manage care for approximately 2,500 patients annually. At present, the center treats about 3,000 radiotherapy courses each year in the radiotherapy department, including referrals from private clinics. Among these patients, approximately 55% are gynecological cancer, while 25% present with breast cancer.

Globally, cervical cancer ranks as the eighth leading cause of death among women [2], with low- and middle-income countries (LMICs) contributing significantly to these numbers being the second most common cause of death for women in the region [4]. Limited access to early diagnosis and treatment in LMICs, along with a shortage of radiation oncology professionals, further exacerbates the issue. In LMICs, professional opportunities in radiation oncology specialties are limited due to insufficient facilities supporting these career paths. The national infrastructure of radiation oncology centers is inadequate to meet population needs; for example, there may be only one radiotherapy machine available for every 3-10 million people. Mortality rates from cervical cancer are three to seven times higher in Latin America and the Caribbean compared to North America (the United States and Canada), as reported by both current and previous Pan American Health Organization (PAHO) assessments [5].

External beam radiation therapy (EBRT) involves creating treatment plans tailored to each patient's unique anatomy to address the disease while maximizing therapeutic benefit. If standardized procedures are not used, developing these personalized plans can be very time-consuming, significantly affecting plan quality while limiting access to treatment. Many radiotherapy centers in LMICs are acquiring advanced, high-precision LINACs and treatment planning systems, but lack adequate training, knowledge, and workflow management for these technologies. These new technologies enable therapeutic dose coverage of the planning target volume (PTV) while protecting surrounding organs at risk (OARs). Traditionally, 3D conformal radiation therapy (3DCRT) with a four-field box technique has been widely used for gynecological cancers, even in advanced cases involving inguinal and paraaortic nodes. While 3DCRT provides consistent dose coverage of the PTV, it significantly compromises sparing of important OARs like the bladder and rectum. Modern inverse planning techniques such as volumetric modulated arc therapy (VMAT) offer improved target coverage, better dose conformity, and enhanced OAR protection [6].

The radiotherapy field in LMICs also faces significant challenges due to insufficient training of medical physicists and dosimetrists in advanced methodologies, including machine quality control, patient-specific quality assurance, and contemporary inverse treatment planning techniques. The process of VMAT planning requires multiple iterations, demanding considerable time and expertise. Achieving an optimal plan that meets clinical objectives requires substantial experience and proficiency. Nelms et al. have demonstrated that significant variability exists among planners, even within a single institution, as individual planners employ different optimization techniques, parameters, priorities, objectives, and methods to delineate optimization volumes for OARs [6].

Knowledge-based planning (KBP) offers an effective solution for creating consistent treatment plans and ensuring consistently high plan quality. KBP involves using planning software that relies on a database of prior patient plans that have been clinically approved and employing these plans as a knowledge base to build models linking geometric characteristics and dosimetry from those cases to forecast achievable dose distributions for new patients [7-9]. After constructing the model, optimization objectives must be specified, which may involve manually or automatically adjusting parameters to generate plans for new patients. This process is demanding, as it requires all selected plans to have been developed using a consistent methodology, to utilize standardized terminology, to follow identical optimization procedures, and, ideally, to represent a large collection of high-quality plans. Building this extensive dataset demands significant planning experience, and a lack of expertise with VMAT can be a considerable obstacle in developing a robust KBP model.

KBP is a well-established solution for radiotherapy centers managing high patient volumes and has demonstrated promising outcomes in clinical planning [10-14]. KBP streamlines the planning workflow by automatically generating dose predictions and applying standardized dose-optimization parameters [15-17]. We hypothesized that KBP would maintain equivalent dosimetric quality to manually generated plans while significantly reducing planning time. This study aimed to evaluate the feasibility of adapting a KBP model developed at a high-income institution for clinical use in an LMIC setting. The primary endpoint was planning efficiency, measured as the plan quality efficiency score (total dosimetric score divided by planning time), with plan quality and OAR sparing evaluated as secondary endpoints.

## Materials and methods

The Instituto Nacional de Cancerología (INCAN) center in Guatemala commissioned a new ring-gantry Halcyon LINAC in 2018 with support from Washington University in St. Louis for clinical setup, workflow development, and staff training. Previous publications from our group have reported the commissioning of the LINAC and the planning to deliver modulated beams, such as VMAT [18,19]. The delivery efficiency of the ring-gantry system was critical to its selection, enabling nearly 90 patients per day to receive radiation therapy over a 14-hour schedule. Gynecological cases at LNCC account for over 50% of the annual patient volume, with more than 3,000 radiotherapy courses delivered each year. Although this study is centered on gynecologic cases, the expertise gained has facilitated the adoption of innovative treatment techniques for additional anatomical sites, including the breast and chest wall. 

The medical physics department at Washington University developed a KBP model utilizing data from over 150 patients, adhering closely to all recommendations established by the KBP originators. These guidelines emphasize standardization throughout the planning process, including consistent contouring of OARs according to established protocols, uniformity in plan generation, and achieving appropriate dose coverage while meeting constraint values. Early access to KBP methodologies was provided by Washington University physicists, which enabled us to apply their model to several patients retrospectively. However, full standardization had not yet been implemented at INCAN, in some activities like the volume contouring. But actually, the standardization is almost completed in all the radiotherapy planning processes.

This retrospective study used only fully anonymized data and did not involve any direct clinical intervention or patient interaction. According to LNCC guidelines, formal IRB approval was not required for this type of secondary analysis. All procedures were performed to ensure the confidentiality and privacy of the source data.

**Figure 1:**
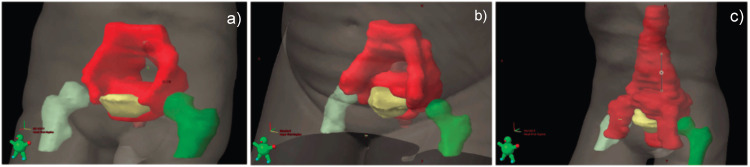
Target volume geometry groups: (a) cervix simple, (b) cervix + inguinal, and (c) cervix + paraaortic Image Credits: Milton E. Ixquiac Cabrera (generated with Eclipse V18, Varian-Siemens Healthineers, Palo Alto, CA, USA).

A key objective of this study is to evaluate the KBP model's ability to meet planning goals and produce clinically acceptable plans, even in the absence of standardized contouring practices and despite significant clinical variability in target volumes observed at INCAN. To this end, we adapted the KBP model for application to INCAN patient data. The shared KBP model was subsequently modified by adding 118 INCAN cases, each reflecting local characteristics such as volume variations and non-standardized clinical contouring. Table 1 outlines all the model structures along with the final modified objectives that were used for planning all cases. These 118 cases were categorized into three groups based on PTV geometry: the first group included only the pelvic volume (which could encompass the cervix and uterus), the second included the pelvic volume plus inguinal nodes, and the third encompassed the previous volumes along with the paraaortic nodes. Figure 1 summarizes the three PTV geometry groups used to categorize the 118 INCAN cases, along with the corresponding arc and isocenter strategies applied during planning.

**Table 1:** Model structures and objectives Optimization constraints for the pelvis gynecologic plans. "Generated" refers to objectives automatically determined by the RapidPlan algorithm. gEUD: generalized equivalent uniform dose

Structure ID	Constraint	Volume (%)	Dose (%)	Priority	gEUD a
PTV_50Gy	Upper	0	102	180	
	Lower	100	100	180	
	Target gEUD		100	180	-40
Z_PTV_50Gy	Upper	0	102	180	
	Lower	100	100	180	
	Target gEUD		100	180	-40
CTV_High	Upper	0	101	120	
	Lower	100	100	130	
	Target gEUD		100.5	120	-40
CTV_Low	Upper	0	101	120	
	Lower	100	100	130	
	Target gEUD		100.5	100	-40
Bladder	Mean		Generated	95	
	Upper gEUD		Generated	95	1
	Line (preferring target)	Generated	Generated	95	
Body	Upper	0	104	200	
Bowel Bag	Line (preferring target)	Generated	Generated	Generated	
Femur	Line (preferring target)	Generated	Generated	Generated	
Kidney	Mean		Generated	Generated	
	Line (preferring target)	Generated	Generated	Generated	
Liver	Line (preferring target)	Generated	Generated	Generated	
NS_Control	Mean		70	Generated	
	Upper gEUD		Generated	Generated	
	Line (preferring target)	Generated	Generated	Generated	
Rectum	Mean		Generated	95	
	Upper gEUD		Generated	95	1
	Line (preferring target)	Generated	Generated	95	
Spinal Cord	Upper	Generated	45	Generated	
	Upper gEUD		40	Generated	40
Uterus	Line (preferring target)	Generated	Generated	Generated	

This study involved a group of four dosimetrists who produced manual treatment plans using both their standard protocols, and an additional plan was created utilizing the KBP model for a validation cohort of 25 gynecological patients. Junior dosimetrists had less than two years of VMAT planning experience, while senior dosimetrists had more than five years. All planners used the same clinical protocol template configured in the treatment planning system. The planning scorecard metric methodology [20] was employed to compare the five plans per patient (four dosimetrists and RapidPlan (Varian-Siemens Healthineers, Palo Alto, CA, USA)). Scorecards are increasingly common plan-evaluation tools that facilitate the assessment of how well a plan adheres to specified dose-volume constraints and simplify interpretation by providing a single numerical measure of plan quality. However, scorecards are not tailored to a patient's individual clinical goals, which at times may require deviations from standard clinical constraints. Nonetheless, in this study, scorecards offered an optimal approach for establishing a consistent metric to compare plans generated by different planners, thereby capturing overall plan quality across all anatomical structures. Comparisons were conducted using a publicly available open-source scorecard metrics Eclipse Scripting Application Programming Interface (ESAPI) plugin designed for Eclipse users, accessible via the Varian Medical Affairs website [21].

Planning

Every plan followed a clinical protocol template configured in the treatment planning system, which contained all necessary information for plan creation. For simple cervical targets and cervix cases with inguinal nodes, we apply three full arcs using different collimator angles (340, 20, and 70 degrees). When treating more complex cervical cases with both inguinal and paraaortic nodes, five full arcs are used: two isocenters are automatically set, one for the upper volume covering the paraaortic region and another for the lower cervical region. Collimator settings for the upper area are 340 and 20 degrees, while those for the lower region are 330, 30, and 90 degrees. For all validation plans, Patient Specific Quality Assurance was conducted using Portal Dosimetry. Each plan was delivered on the LINAC and evaluated using Gamma criteria of 3%/2 mm to confirm the feasibility of delivery.

Comparison

The evaluation of the KBP plan and other planning methods was conducted using the Dosimetric ScorePlan, adapted from the Varian Medical Affairs examples. The results were presented graphically to illustrate variability among the plans. Subsequent adjustments were made to enhance the model, including modifications to certain constraints to improve optimization and the inclusion of additional patient data to increase robustness. The final step toward implementing the RapidPlan model involved validation across 25 cervical treatment cases. The KBP model was applied to these cases to assess metrics such as Conformity Index, D0.03cc, V47.5 Gy, and V49 Gy for the PTV; V40 Gy and V45 Gy for the rectum and V30 Gy and V40 Gy for the bladder; and V30 Gy for the femoral heads. Finally, a plan quality efficiency score was defined as the total plan score divided by the total planning time in minutes. This metric allowed a comparison of plan quality relative to the time required to generate the plan. Table 2 lists the scorecard metric thresholds used to compute plan scores for targets and OARs.

**Table 2:** Scorecard metric values and thresholds used for plan evaluation

Volume	Metric	Threshold (%)	Score	Metric	Threshold (%)	Score	Metric	Threshold (Gy)	Score
PTV50_Gy_	V49_Gy_	≤85	0	V45_Gy_	≤97	0	D_2%_	≤48	12
		≤90	3		≤98	3		≤54	9
		≤95	9		≤99	9		≤58	3
		≤98	12		100	12		≤62	0
Bladder	V30_Gy_	≤35	6	V40_Gy_	≤15	6			
		≤43	4.5		≤25	4.5			
		≤50	3.75		≤35	3.75			
		≤60	0		≤52	0			
Rectum	V40_Gy_	≤37.5	12	V45_Gy_	≤20	12			
		≤45	10		≤25	9			
		≤55	5		≤30	6			
		≤60	0		≤40	0			
Femoral heads	V30_Gy_	≤1	2						
		≤5	1.5						
		≤10	1						
		≤20	0						

## Results

**Figure 2:**
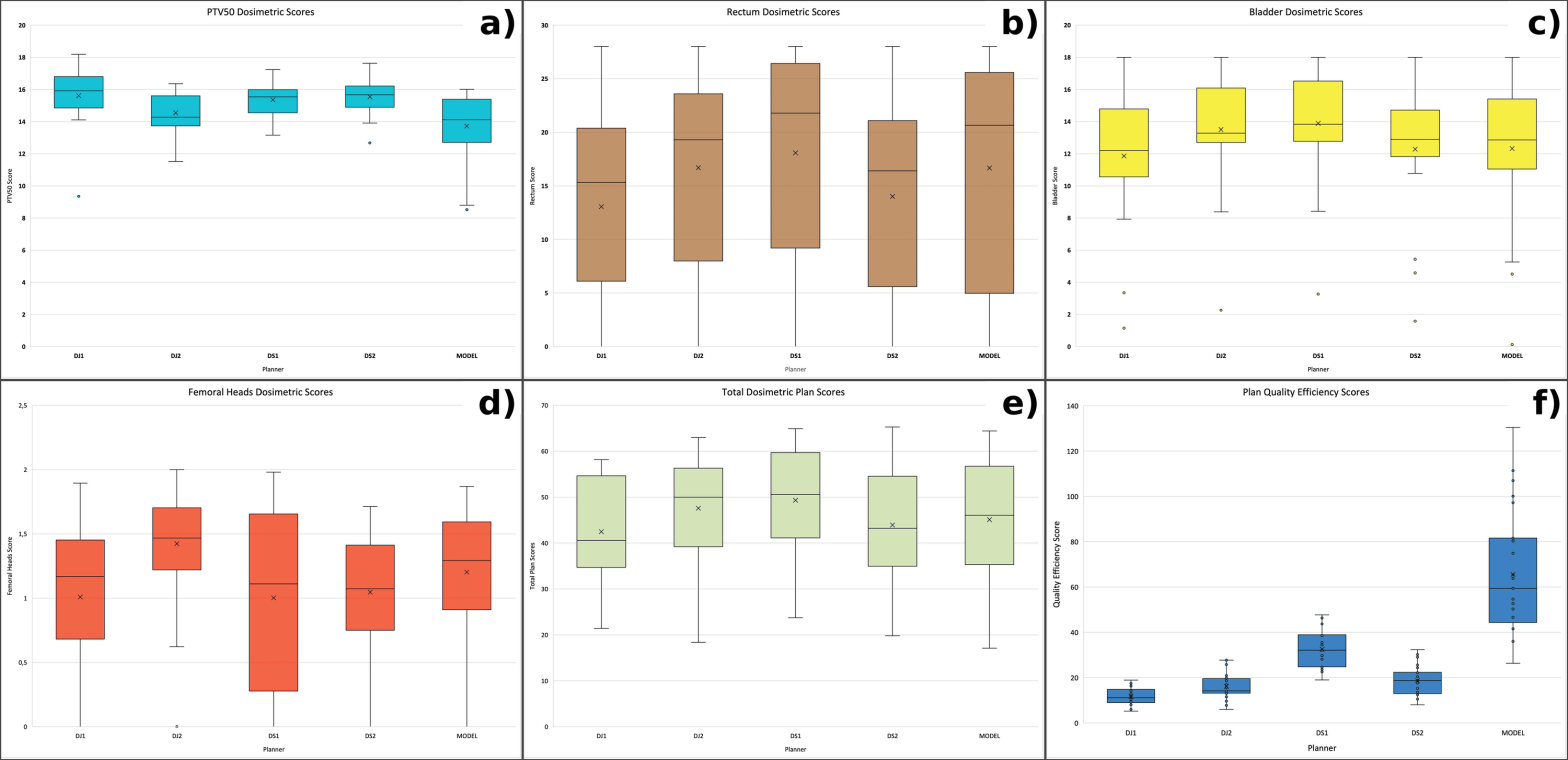
Distribution of dosimetric scores for target (a) PTV50 and various OARs (b) rectum, (c) bladder, and (d) femoral heads, (e) total dosimetric plan scores, and (f) plan quality efficiency score OARs: organs at risk Image Credits: Milton E. Ixquiac Cabrera (generated with R (R Foundation for Statistical Computing, Vienna, Austria))

**Figure 3:**
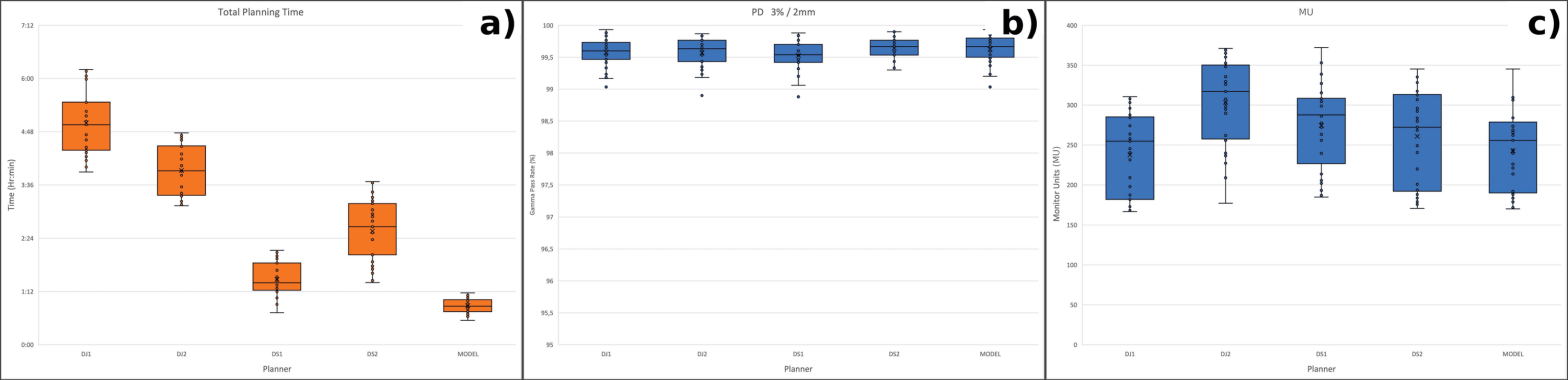
Distribution of (a) total planning time, (b) portal dosimetry results global per planner, and (c) MU needed for each planner MU: monitor units Image Credits: Milton E. Ixquiac Cabrera (generated with R (R Foundation for Statistical Computing, Vienna, Austria)).

The dosimetric scores for PTV50 (Figure 2a), rectum (Figure 2b), bladder (Figure 2c), and femoral heads (Figure 2d), total dosimetric plan scores (Figure 2e), and plan quality efficiency scores (Figure 2f) were compared using box-whisker plots to get an overview of the distribution of scores in Figure 2. Each figure shows the upper/lower quartiles, the median, and the upper/lower extremes for each group. The total planning time (Figure 3a), gamma passing rates (Figure 3b), and total monitor units (MU) (Figure 3c) are also shown in Figure 3 using box-whisker plots. Across planners and planning approaches, overall plan quality was consistent, with no systematic differences between manually generated plans and model-generated plans. This similarity likely reflects the use of well-defined clinical goals and a standardized plan-review process that promotes consistency regardless of the planner or planning method. Despite comparable plan quality, the model reduced plan creation time, indicating that similar-quality plans can be generated more efficiently. Across evaluated structures, the largest improvement was observed in rectum-related dose metrics, where the model achieved higher scores than the manual plans. Overall, these results suggest the model maintains clinical plan quality while improving planning efficiency and providing incremental benefit for selected OARs.

Gamma analysis (3%/2 mm) (Figure 3b) was used to assess delivery feasibility, and all plans achieved similar passing rates (mean 99.6%). MU were also comparable across plans, with mean MU by planner of 238, 305, 275, 260, and 245; the overall mean was 264 MU with a standard deviation of 57 MU. Modulation and complexity indices were similarly consistent across planners.

## Discussion

This study demonstrates that KBP can substantially improve the efficiency of radiotherapy treatment planning while maintaining high, consistent plan quality in an LMIC setting. Radiotherapy services in LMICs face well‑documented structural constraints, including limited numbers of treatment machines, shortages of trained radiation oncology professionals, and high patient volumes [1,2]. In this context, time-intensive manual treatment planning can become a bottleneck, delaying treatment and placing additional strain on already-limited human resources. The ability to generate clinically acceptable plans more efficiently has direct implications for patient access, system sustainability, and consistency of plan quality [2].

The results of this work show that plans generated with KBP achieved comparable overall plan quality to manually generated plans across multiple planners, without evidence of systematic degradation in target coverage or OAR sparing. This finding is particularly relevant in LMIC settings, where variability in planner experience and training can contribute to inconsistent plan quality [6]. By incorporating prior clinical knowledge into the planning process, KBP reduces reliance on individual planner expertise and helps standardize plan quality across users [8,9], even in the presence of heterogeneous clinical practices and non‑uniform contouring [6]. The observed improvements in rectal dose metrics further suggest that KBP may not only preserve quality but also provide incremental benefits for selected OARs. Improved rectal sparing may translate clinically into reduced rates of late gastrointestinal toxicity, which is particularly relevant in LMIC settings where supportive care resources for managing side effects are also limited.

A key contribution of this study is the demonstration that KBP models developed in high‑income countries can be adapted for effective use in LMIC environments. KBP has demonstrated promising outcomes across diverse anatomical sites and institutional contexts [10-14]. Importantly, the adapted model remained robust despite significant variability in target volumes and contouring practices, which are common challenges in centers with rotating physician staff and evolving clinical protocols. This adaptability lowers the barrier to KBP implementation in resource‑limited settings, where achieving full standardization before model deployment may not be feasible. Rather than requiring ideal conditions, the approach described here supports incremental improvement, allowing centers to realize efficiency gains while continuing to mature their clinical workflows [16,17].

The findings also underscore the importance of aligning technological adoption with workflow optimization and training [22]. While many LMIC centers have gained access to modern LINACs and treatment planning systems [18,19], the full clinical benefit of these technologies cannot be realized without efficient and reproducible planning processes. KBP offers a practical mechanism to bridge this gap by embedding best practices directly into routine clinical planning, thereby amplifying the impact of limited human expertise. However, it should be noted that successful KBP implementation requires careful planning around practical barriers, including software licensing costs, dedicated staff training, and progressive workflow standardization, factors that must be considered when evaluating the feasibility of adoption in resource-constrained settings.

Several limitations should be acknowledged. This study was conducted at a single institution and focused on gynecologic cases, which may limit generalizability to other disease sites or practice environments. In addition, ongoing variability in contouring practices likely influenced achievable dose metrics and may have attenuated some advantages of KBP. Future work should include prospective multicenter validation across additional LMIC institutions and extension to other disease sites such as the breast and head and neck, which will be important to further define the role of KBP in LMIC radiotherapy programs.

## Conclusions

The rapid expansion of our center, equipped with modern LINACs and the implementation of image-guided radiation therapy (IGRT) and intensity-modulated radiation therapy (IMRT) techniques, has enhanced the quality of care delivered to our patients. The data demonstrated that integrating KBP into clinical practice has substantially decreased planning times, enabling our physics and dosimetry teams to manage greater patient volumes while devoting additional attention to complex treatment plans. It is important to note that standardizing contours among the rotating physician group remains an ongoing effort at our center. Variability in contouring may affect the consistency of achievable dose metrics and potentially obscure some advantages associated with KBP. These findings support KBP as a practical strategy to improve planning efficiency in resource‑constrained settings, pending validation across additional institutions and disease sites.
